# The importance of modeling the human cerebral vasculature in blunt trauma

**DOI:** 10.1186/s12938-021-00847-x

**Published:** 2021-01-14

**Authors:** Dhananjay Radhakrishnan Subramaniam, Ginu Unnikrishnan, Aravind Sundaramurthy, Jose E. Rubio, Vivek Bhaskar Kote, Jaques Reifman

**Affiliations:** 1 Department of Defense Biotechnology High Performance Computing Software Applications Institute, Telemedicine and Advanced Technology Research Center, United States Army Medical Research and Development Command, FCMR-TT, 504 Scott Street, Fort Detrick, MD 21702-5012 USA; 2 The Henry M. Jackson Foundation for the Advancement of Military Medicine, Inc, 6720A Rockledge Drive, Bethesda, MD 20817 USA

**Keywords:** Blunt impact, Traumatic brain injury, Finite element model, Human cerebral vasculature

## Abstract

**Background:**

Multiple studies describing human head finite element (FE) models have established the importance of including the major cerebral vasculature to improve the accuracy of the model predictions. However, a more detailed network of cerebral vasculature, including the major veins and arteries as well as their branch vessels, can further enhance the model-predicted biomechanical responses and help identify correlates to observed blunt-induced brain injury.

**Methods:**

We used an anatomically accurate three-dimensional geometry of a 50th percentile U.S. male head that included the skin, eyes, sinuses, spine, skull, brain, meninges, and a detailed network of cerebral vasculature to develop a high-fidelity model. We performed blunt trauma simulations and determined the intracranial pressure (ICP), the relative displacement (RD), the von Mises stress, and the maximum principal strain. We validated our detailed-vasculature model by comparing the model-predicted ICP and RD values with experimental measurements. To quantify the influence of including a more comprehensive network of brain vessels, we compared the biomechanical responses of our detailed-vasculature model with those of a reduced-vasculature model and a no-vasculature model.

**Results:**

For an inclined frontal impact, the predicted ICP matched well with the experimental results in the fossa, frontal, parietal, and occipital lobes, with peak-pressure differences ranging from 2.4% to 9.4%. For a normal frontal impact, the predicted ICP matched the experimental results in the frontal lobe and lateral ventricle, with peak-pressure discrepancies equivalent to 1.9% and 22.3%, respectively. For an offset parietal impact, the model-predicted RD matched well with the experimental measurements, with peak RD differences of 27% and 24% in the right and left cerebral hemispheres, respectively. Incorporating the detailed cerebral vasculature did not influence the ICP but redistributed the brain-tissue stresses and strains by as much as 30%. In addition, our detailed-vasculature model predicted strain reductions by as much as 28% when compared to current reduced-vasculature FE models that only include the major cerebral vessels.

**Conclusions:**

Our study highlights the importance of including a detailed representation of the cerebral vasculature in FE models to more accurately estimate the biomechanical responses of the human brain to blunt impact.

## Introduction

The Centers for Disease Control and Prevention (CDC) has identified traumatic brain injury (TBI) as one of the major causes of mortality in the U.S., annually contributing to nearly 30% of all injury-related deaths [1]. In a 2013 study, the CDC reported approximately 2.8 million cases of TBI resulting from motor vehicle accidents, falls, and other blunt loads to the head, including 282,000 TBI-related hospitalizations and 56,000 deaths [2]. Mechanical forces to the head, such as those caused by a blunt impact (i.e., a direct blow to the head) or an impulse (i.e., a sudden movement of the head) can potentially cause TBI [3]. Impacts and impulses are dynamic inertial loads that act either normal or tangential to the head. Normal impacts occur less frequently and generate only translation of the head. In contrast, tangential impacts occur frequently in motor vehicle accidents and falls, and cause both translation and rotation of the head [4]. Translation of the head resulting from a normal or tangential impact can potentially increase the pressure in the head [3], which is hypothesized to cause focal injuries depending on the maximum pressure experienced by the head over the duration of the blunt impact. In contrast, rotation of the head resulting from a tangential impact possibly causes shear deformation of the brain tissue, which can subsequently damage the axonal fibers embedded in the white matter and cause diffuse injuries [4].

One way to assess the risk of TBI due to blunt trauma is to use computational models to predict the biomechanical responses of the head to the blunt loading and correlate these responses with observations of brain damage. For instance, to identify potential injury thresholds for TBI, two separate research groups have independently developed three-dimensional (3-D) finite element (FE) models of the human head and correlated the simulated responses with injuries observed in computed tomography images [5, 6]. Moreover, to evaluate the biomechanical response of the brain to impacts and impulses that generate translation and rotation of the head, different research groups have developed 3-D FE models of the human head, which vary greatly in terms of the number of anatomical components, material properties of the brain tissue, brain anatomy, and description of the cerebral vasculature [7–22]. For example, Tse et al. included 13 components in their FE model [20], whereas the FE model developed by Cotton et al. consisted of 32 components [8]. Although the model developed by Cotton et al. contributed toward improving the anatomical description of the human head, the material properties of the brain were obtained from bovine brain tissue specimens, which could possibly limit the accuracy of the predicted biomechanical response. To address this limitation, other studies [23, 24] modeled the brain using material properties obtained from mechanical testing of human brain tissue [25, 26]. The human brain comprises over 643,738 m of vasculature, including arteries, veins, arterioles, and venules [27]. As such, Zhang et al. [28] as well as Zhao and Ji [29] showed that the inclusion of the cerebral vessels in a FE model of the human head stiffens the brain tissues, decreasing brain-tissue strain. Moreover, Ho and Kleiven [30] showed that in addition to the major cerebral veins, inclusion of the major cerebral arteries reduces the brain-tissue strain by as much as 8% for blunt loading. However, exclusion of the branch vessels limited the accuracy of the predicted strain.

We hypothesize that inclusion of a more comprehensive network of cerebral veins and arteries, including precise distinction between the superficial vessels that conform to the brain surface convolutions and the internal vessels embedded deep within the brain tissue, can further enhance the accuracy of the biomechanical responses of the human head to blunt trauma. To this end, we developed an anatomically accurate 3-D FE model of the human head that includes such a detailed network of cerebral veins and arteries. We simulated the biomechanical responses of the human brain to (1) an inclined frontal impact, (2) a normal frontal impact, and (3) an offset parietal impact. Next, we quantified the influence of the cerebral vasculature by comparing the biomechanical responses of our detailed-vasculature model with those of a reduced-vasculature model and a no-vasculature model.

## Materials and methods

### Head model geometry

**Fig. 1:**
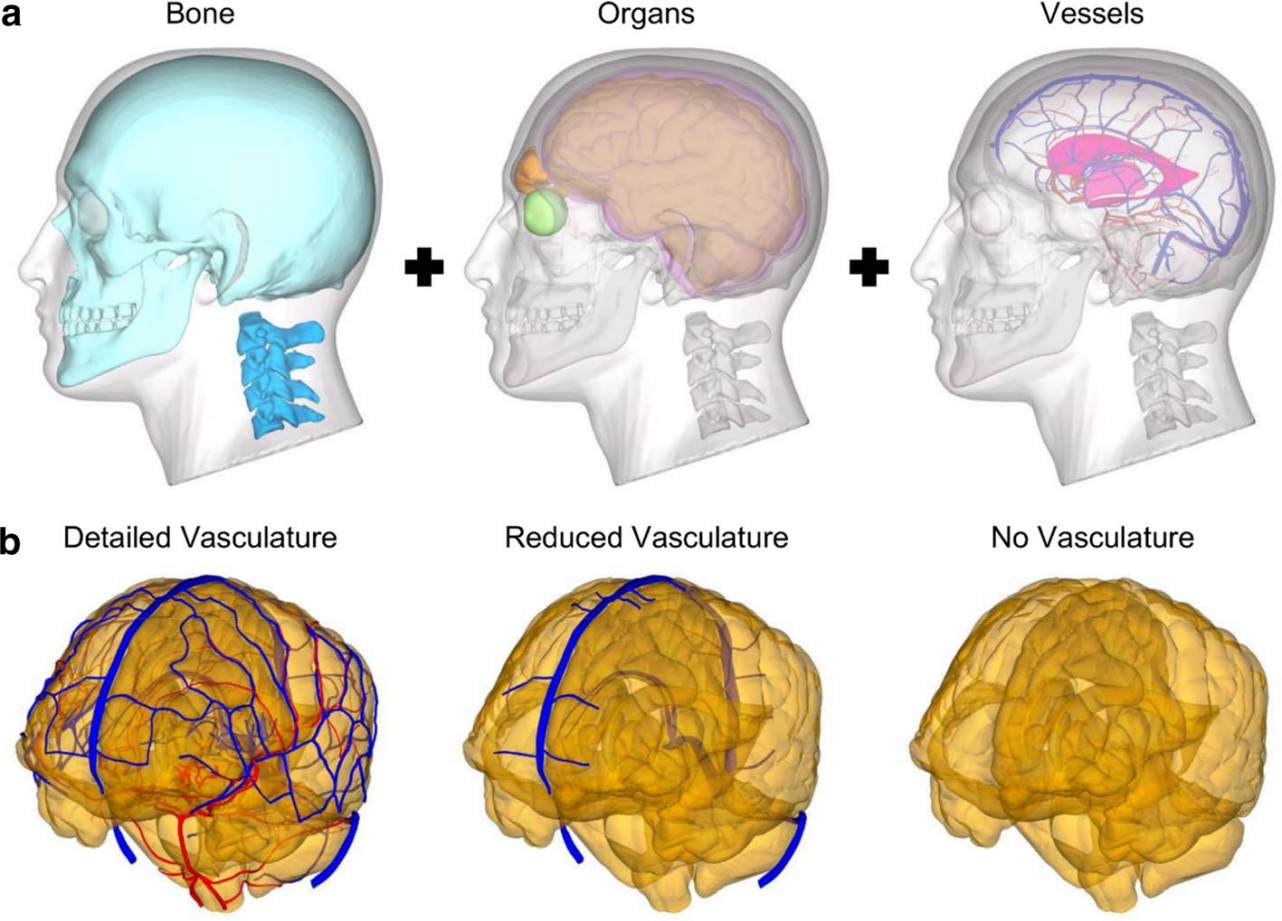
Head geometry and vasculature models. **a** Bone (skull, cyan; cervical spine, light blue), organs (brain, gold; subarachnoid space, purple; eyes, green; frontal sinus, orange; ventricle, magenta), and vasculature (arteries, red; veins, dark blue) included in the high-fidelity finite element model. **b** Comparison between the detailed-vasculature, reduced-vasculature, and no-vasculature models (Note: skin and adipose tissue are displayed with a transparent color)

We acquired an anatomically accurate 3-D geometry of a 50th percentile U.S. male head from Zygote Media Group, Inc. (American Fork, UT). The geometrical model, generated using medical atlases, comprised the skin, eyes, sinuses, cervical spine, skull, brain, meninges, superficial veins, and superficial arteries (Fig. 1a). The superficial veins comprised the superior and inferior sagittal sinus, sigmoid sinus, transverse sinus, straight sinus, occipital sinus, great cerebral vein, cerebellar veins, and the detailed network of cerebral veins. The superficial arteries comprised the basilar artery, vertebral artery, superior and inferior cerebellar arteries, anterior cerebral arteries, middle cerebral arteries, anterior communicating artery, and the detailed network of posterior communicating arteries. Using publicly available whole-brain venous and arterial voxel-based probabilistic atlases previously acquired using multi-band time-of-flight angiography and high-resolution multi-echo susceptibility-weighted magnetic resonance imaging [31], we reconstructed additional veins and arteries embedded within the brain tissue. We used the thresholding algorithm in 3D Slicer 4.10 [32] to segment the internal vein, posterior fossa veins, deep middle cerebral veins, and lenticulostriate arteries. We then registered the geometry of the embedded vasculature with the brain of the proposed head model. The cerebral vasculature in our model included the superficial and embedded veins (minimum diameter of 0.52 mm), the arteries (minimum diameter of 0.24 mm), and their branch vessels, whose total vasculature length evaluated using VMTK 1.4 [33] amounted to 15 m. To quantify the influence of including the detailed network of vessels, we developed two additional models (Fig. 1b): (1) a reduced-vasculature model [8, 16] that consisted of the sagittal sinus, sigmoid sinus, transverse sinus, straight sinus, occipital sinus, great cerebral vein, and a truncated network of cerebral veins (total vasculature length of 2 m) and (2) a no-vasculature model.

### Geometry processing and mesh generation

**Fig. 2:**
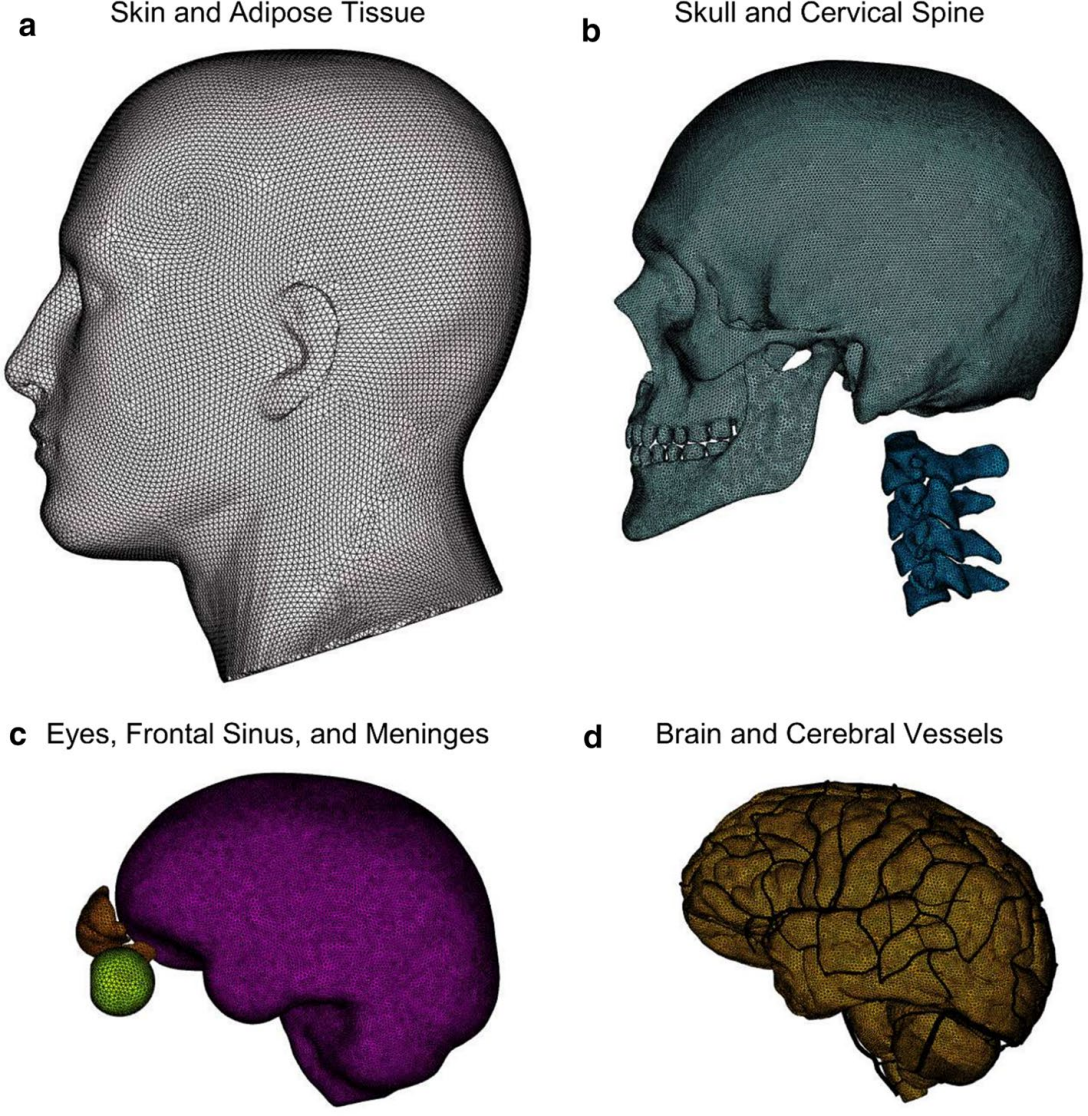
Finite element mesh of the human head. **a** Skin and Adipose Tissue. **b** Skull and Cervical Spine. **c** Eyes, Frontal Sinus, and Meninges. **d** Brain and Cerebral Vessels

We processed the polygon geometries corresponding to the bone, organs, skin, and vasculature using Blender 2.79 [34] and used the software to remove geometric artifacts, small features, and interferences between different components. We meshed the polygon geometries using OpenFlipper 3.1 [35], by creating triangular surface meshes of individual components without the loss of important anatomical features. We evaluated the surface meshes of individual components using MeshLab 2016 [36] for manifoldness and ensured a watertight mesh. To evaluate the quality of the watertight mesh, we exported it to HyperMesh 2017.1 (Altair Engineering, Troy, MI) and ensured that there were no warped or highly skewed elements by repairing meshes of inferior quality and re-triangulating them. We integrated the triangular meshes corresponding to the skin, eyes, sinuses, cervical spine, skull, brain, and meninges using the mesh Boolean operations available in CloudCompare 2.10 [37]. We generated tetrahedral volume meshes of the aforementioned anatomical components using HyperMesh (total number of tetrahedral elements: 4,289,775). Next, we converted the linear tetrahedrons to modified quadratic tetrahedrons (C3D10M) [38] and merged the volume meshes to prevent relative motion between different anatomical components. We converted the vasculature surface mesh to reduced-integration (S3R) shell elements (total number: 825,898). The FE mesh consisted of elements having an average length of 2.4 mm for the skin and adipose tissue, 2.2 mm for the skull and cervical spine, 2.0 mm for the eyes, frontal sinus, and meninges, 2.3 mm for the brain, and 0.27 mm for the vasculature (Fig. 2a–d). Based on previous geometric measurements of the human cerebral vessel wall [39], we assigned shell thicknesses of 0.12 mm and 0.10 mm to the veins and arteries. We used the embedded element method [9] to enforce a no-slip condition between the superficial vasculature and the subarachnoid space and between the internal vasculature and the brain.

### Material properties

We obtained the material properties of different human anatomical components from the literature. We used material properties obtained from mechanical tests performed on post-mortem brain-tissue samples for the brain [40]; material properties obtained from mechanical tests performed on freshly excised cortical veins and arteries for the human cerebral vasculature [39]; and material properties obtained from mechanical tests performed on post-mortem skin-tissue samples for the human skin [41]. We modeled the skin, veins, and arteries using a one-term Ogden model and assumed that the material properties of connective tissues were the same as those for the skin [42]. We modeled the deviatoric response of the brain using a Mooney-Rivlin model and a two-term Prony series [25] and the eyes, ventricles, and subarachnoid space each as neo-Hookean solids [8, 43, 44]. For material properties of the meninges, we used values within the range of those employed in previous computational studies [28, 45]. Finally, we modeled the volumetric response of the soft-tissue components using a high bulk modulus [23, 45], represented the frontal sinus using the equation of state for air at atmospheric pressure [8], and modeled the skull and vertebrae as linear elastic materials. For the material parameters of bone, we used values within the range of those employed in previous computational studies [19, 38, 46, 47]. Table 1 summarizes the material properties for the different anatomical components of the human head FE model.

**Table 1:** Summary of the material properties used for the individual anatomical components included in the high-fidelity, detailed-vasculature human head model

**Component**	**Density** (kg/m^3^)	**Elastic Constants**		**Hyperelastic Constants**			**Prony Coefficients**			
		*Elastic Modulus (GPa)*	*Poisson's Ratio*	*Bulk Modulus (GPa)*	*Shear Modulus (kPa)*	*α*	*g*_1_	*g*_2_	*τ*_1_ *(s)*	*τ*_2_ *(s)*
Spine [19, 38, 47]	1412	6.50	0.22							
Skull [19, 38, 47]	1412	6.50	0.22							
Arteries [39]	1040			2.11	898.00	9.49				
Skin [41]	1040			0.04	23,900.00	16.55				
Brain [25, 40]	1040			2.19	2.62		0.63	0.36	0.008	0.15
Veins [39]	1040			2.11	266.00	7.46				
Eyes [43, 44]	1040			2.19	8.00					
Meninges [28, 45]	1040			2.19	1.97					

### Blunt impact experiments

To describe the relationship between different blunt impact variables, Nahum et al. performed head impact experiments on seated cadavers [48]. And to maintain the vascular and cerebrospinal fluid pressure within the normal physiological range, they pressurized the unembalmed cadaver heads. In addition, to prevent skull fracture and vary the impact duration, they used different padding materials between the skull and the impactor. They inclined the cadaver head by an angle of 45° to the Frankfort plane and delivered a blow to the frontal bone with the rigid impactor traveling at a velocity ranging from 4.4 to 13.0 m/s [48]. To quantify the biomechanical responses to the blunt impact, Nahum et al. measured the force exerted by the impactor, the acceleration of the head, and the intracranial pressure values at different locations within the cranium.

To validate their human head FE model, Trosseille et al. performed head impact experiments on seated cadavers [49]. They pressurized the unembalmed cadaver heads, similar to Nahum’s study [48]. Using a 23.4 kg impactor traveling at a velocity of 7.0 m/s, they delivered the blow to the facial region of the cadaver in the anterior–posterior direction [49]. Unlike Nahum’s study, Trosseille et al. aligned the cadaver’s head with the Frankfort plane and performed the tests with and without padding materials. To measure the kinematics of the head, they implanted a 12-accelerometer array in the occipital region of the skull, and to measure the intracranial and ventricular pressures, they placed miniature pressure transducers in the brain and the ventricles, respectively.

To evaluate the motion of the brain relative to the skull, Hardy et al. performed occipital, temporal, and parietal impacts on cadaver heads [50]. They perfused the heads, obtained from unembalmed cadavers, with artificial cerebrospinal fluid at a constant pressure of 10.3 kPa. To deliver the blow to the head, they turned the cadaver upside down to maintain the fluid in the brain, accelerated the head using a pneumatic piston, and then stopped it rapidly against an acrylic block. To generate a large linear acceleration, they aligned the impact location with the center of gravity of the head. In contrast, to generate a large angular acceleration, they offset the impact location from the center of gravity of the head. To measure the kinematics of the head, Hardy et al. mounted a 9-accelerometer array in the face of the cadaver. Next, to image the motion of the brain using a high-speed X-ray device, they implanted neutral density targets (NDTs) and skull markers in the cadaver head [50]. For occipital impacts, they implanted one cluster of NDTs in the frontal lobe and one cluster in the parietal lobe, whereas for temporal and occipital impacts, they implanted one cluster of NDTs in the right cerebral hemisphere and one cluster in the left cerebral hemisphere. Finally, to measure intracranial pressure, they placed cranial pressure transducers at the coup and contrecoup locations.

### Blunt impact simulations

**Fig. 3:**
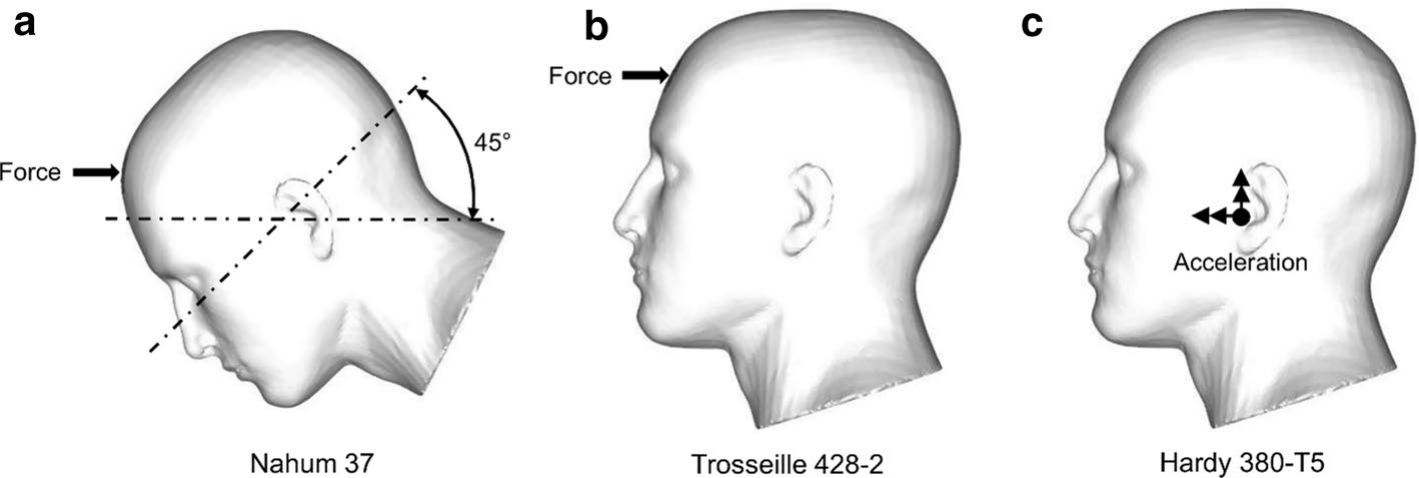
Setup of blunt trauma experiments. **a** Setup of Nahum’s experiment. **b** Setup of Trosseille’s experiment. **c** Setup of Hardy’s experiment

We simulated three blunt loading experiments: (1) Nahum’s experiment 37, which involved a cylinder impacting the human head inclined at 45° to the Frankfort plane [48]; (2) Trosseille’s experiment 428–2, which involved a steering wheel impacting the forehead in the anterior–posterior direction [49]; and (3) Hardy’s experiment 380-T5, which involved an offset left parietal impact [50]. To simulate Nahum’s experiment, we applied the measured impact force on the scalp of the FE model (Fig. 3a) as a pressure force distributed over an area of 1330 mm^2^ [38]. Next, to simulate Trosseille’s experiment, we derived the impact force from the measured acceleration [12] and applied the force on the forehead of the FE model (Fig. 3b) as a pressure force distributed over an area of 1360 mm^2^. Finally, to simulate Hardy’s experiment, we modeled the face using discrete rigid elements [51] and applied the measured linear and angular accelerations to the center of gravity of the head (Fig. 3c) [15, 51]. We did not constrain the neck of the FE model, consistent with previous studies [12, 15, 21, 45]. We performed blunt loading simulations using ABAQUS/Explicit 2018 (Dassault Systèmes Simulia Corp., Johnston, RI) on (1) a SGI 8600 system termed Mustang at the U.S. Air Force Research Laboratory Supercomputing Resource Center, (2) a Cray XC40/50 system termed Onyx at the U.S. Army Engineer Research and Development Center, and (3) a SGI ICE XA system termed Centennial at the U.S. Army Research Laboratory Supercomputing Resource Center.

We post-processed the simulation results using EnSight 10.2.5a (Computational Engineering International, Inc., Apex, NC) and determined the intracranial pressure (ICP), the relative displacement (RD) between the brain and skull, the von Mises stress (VMS), and the maximum principal strain (MPS). We evaluated the ICP because it is a clinically relevant parameter used to determine the balance among the intracranial content volumes (i.e., the brain tissue, cerebrospinal fluid, and cerebral blood) [52]. A blunt impact can change such a balance and increase the ICP [52], generating pressure gradients within the head [53] and subsequently causing brain contusions [54]. The RD, which is a measure of the brain inertial lag, can cause brain contusions as the result of a blunt trauma involving head translation [55], similar to the ICP. In scenarios involving head rotation, the RD can possibly tear the bridging veins and cause subdural hematoma [56]. Moreover, while blunt-induced shear stress can potentially cause neurocellular dysfunction [57], axonal stretch can damage the white matter in the brain [58]. Here, we used the model-predicted values of VMS [7] and MPS [4] as surrogates for blunt-induced shear stress and axonal stretch, respectively. We validated our high-fidelity, detailed-vasculature 3-D FE model by comparing the model-predicted ICP and RD values with experimentally measured values [48–50]. In addition, for Nahum’s experiment, we compared and contrasted the ICP, VMS, and MPS values predicted by the detailed-vasculature model with those predicted by the reduced-vasculature model and the no-vasculature model.

## Results

**Fig. 4:**
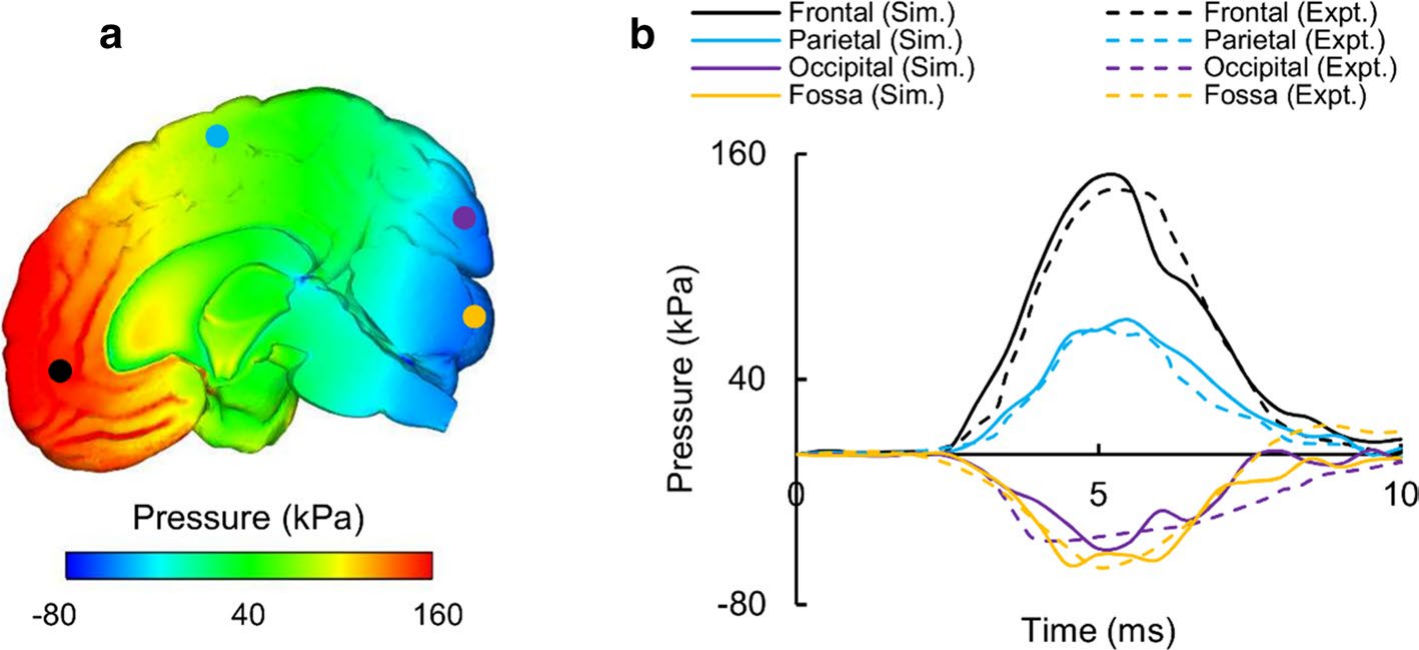
Intracranial pressure (ICP) measured in Nahum’s experiment [48] and simulated with the detailed-vasculature model. **a** Contour map showing the predicted ICP distribution throughout the mid-sagittal brain (t = 5 ms). **b** Magnitude and temporal profile of the simulated (Sim.) and experimentally (Expt.) observed ICP values at the frontal lobe, fossa, parietal lobe, and occipital lobe (Locations selected for comparison are indicated by colored circles on the contour map)

We validated our high-fidelity FE model for Nahum’s experiment by comparing the model-predicted and experimentally measured ICP values at four locations [48]: (1) frontal lobe, (2) parietal lobe, (3) occipital lobe, and (4) fossa. Figure 4a shows the distribution of the predicted ICPs throughout the mid-sagittal brain, whereas Fig. 4b shows the measured and predicted temporal profiles of the ICP values at the aforementioned locations, which showed close correspondence with an overall phase-shift error of 10%. In particular, the coup (i.e., positive ICP at the frontal lobe) and contrecoup (i.e., negative ICP at the posterior fossa) pressures predicted by the detailed-vasculature model matched well with those observed experimentally, with a phase-shift error of 9.1% in the frontal lobe and 10.1% in the posterior fossa. The measured and model-predicted peak ICP values also matched well, with differences of 5.3% at the frontal lobe, 6.2% at the parietal lobe, 2.4% at the posterior fossa, and 9.4% at the occipital lobe. Comparisons of the reduced- and no-vasculature models with the detailed-vasculature model revealed no differences in the magnitude or time course of the simulated ICP.

**Fig. 5:**
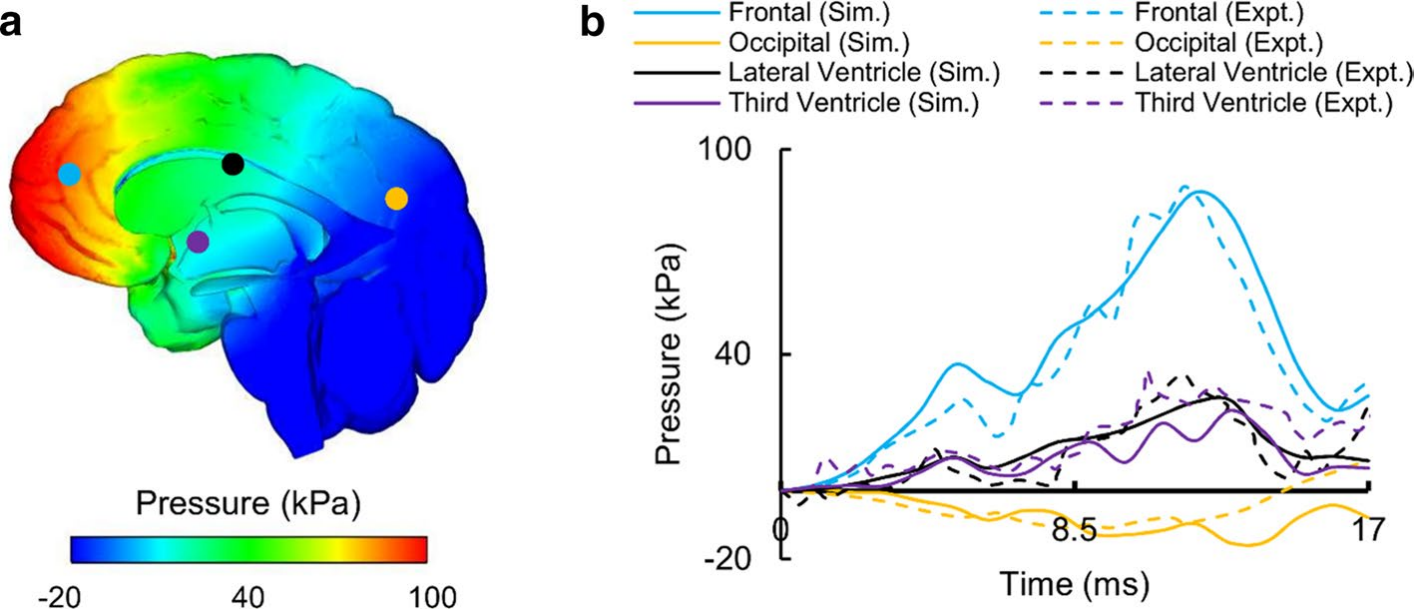
Intracranial pressure (ICP) measured in Trosseille’s experiment [49] and simulated with the detailed-vasculature model. **a** Contour map showing the predicted ICP distribution throughout the mid-sagittal brain (t = 12 ms). **b** Magnitude and temporal profile of the simulated (Sim.) and experimentally (Expt.) observed ICP values at the frontal lobe, occipital lobe, lateral ventricle, and third ventricle (Locations selected for comparison are indicated by colored circles on the contour map)

Next, we validated our high-fidelity FE model for Trosseille’s experiment by comparing the model-predicted and experimentally measured ICP values at four locations [49]: (1) frontal lobe, (2) occipital lobe, (3) lateral ventricle, and (4) third ventricle. Figure 5a shows the distribution of the predicted ICPs throughout the mid-sagittal brain, whereas Fig. 5b shows the measured and predicted temporal profiles of the ICP values at the aforementioned locations. In particular, the frontal and lateral ventricle pressures predicted by the detailed-vasculature model matched well with those observed experimentally, with a phase-shift error of 2.6% in the frontal lobe and 11.6% in the lateral ventricle. The measured and model-predicted peak ICP values also matched well with differences of 1.9% at the frontal lobe and 22.3% at the lateral ventricle. In contrast, the ICP magnitude and phase-shift differences were equivalent to 40.7% and 36.1%, respectively, at the occipital lobe and 33.7% and 22.9%, respectively, at the third ventricle.

**Fig. 6:**
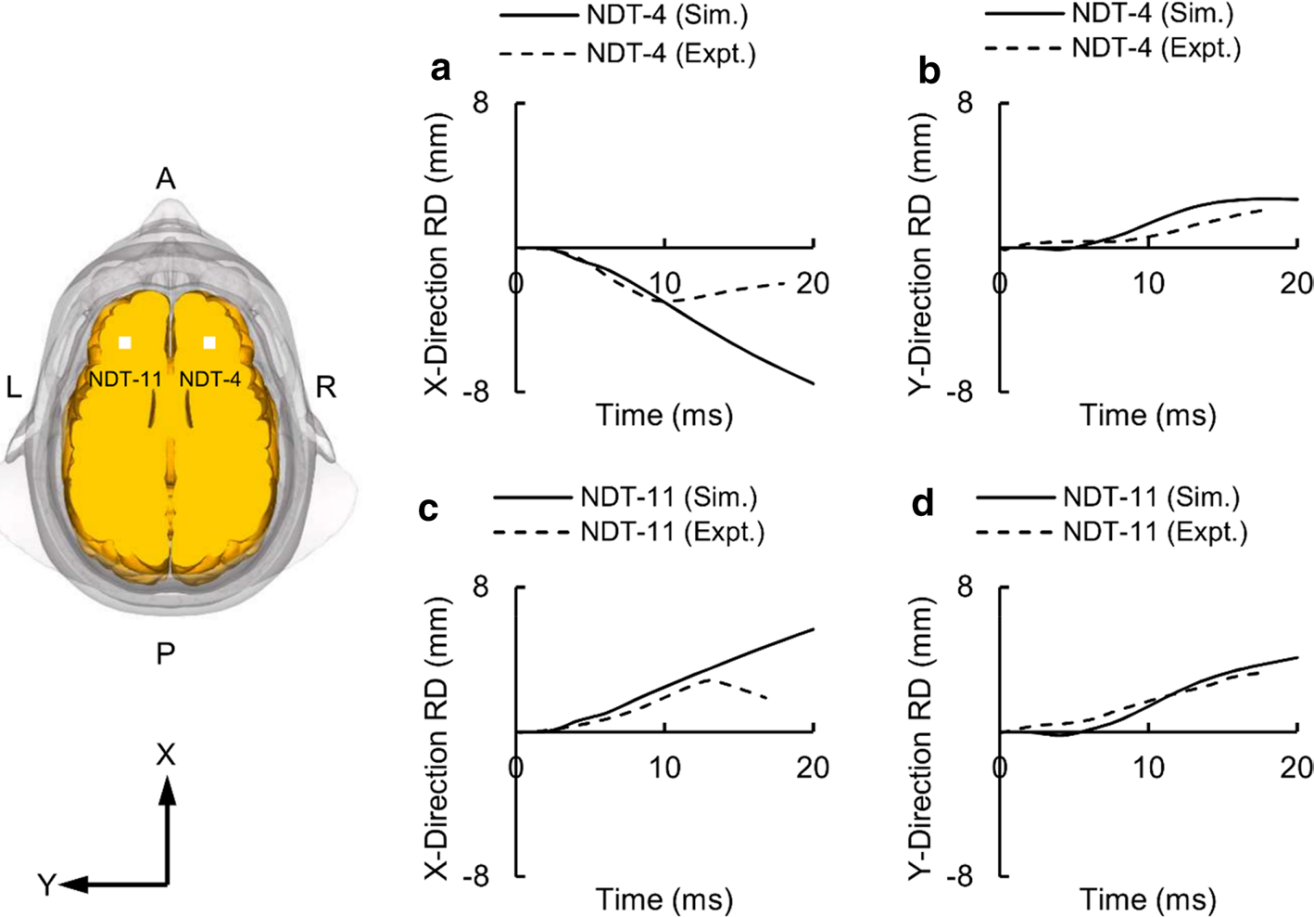
Relative displacement (RD) measured in Hardy’s experiment [50] using neutral density targets (NDT) and simulated with the detailed-vasculature model. Magnitude and temporal profile of the simulated (Sim.) and experimentally (Expt.) observed RD values corresponding to **a** NDT-4 in the X direction, **b** NDT-4 in the Y direction, **c** NDT-11 in the X direction, and **d** NDT-11 in the Y direction (Locations of NDT-4 and NDT-11 are indicated by white squares on the brain geometry. A: anterior; P: posterior; R: right; L: left)

Finally, we validated our high-fidelity FE model for Hardy’s experiment by comparing the model-predicted and experimentally measured RD values. Specifically, we compared the X and Y components of the model-predicted RD values with the corresponding experimental measurements for NDT-4 located in the right cerebral hemisphere and NDT-11 located in the left cerebral hemisphere [50]. Initially (i.e., from 0 to 10 ms), the RD of the model-predicted X component increased monotonically with time and matched well with the experimental measurements (Fig. 6a, c). However, after this initial phase, the experimentally measured values decreased with time, whereas the model-predicted values increased with time. The RD of the model-predicted Y component was slightly delayed (Fig. 6b, d). However, it started to increase after 4 ms, matching well with the experimental measurements, with maximum RD differences of 27% in the right cerebral hemisphere and 24% in the left cerebral hemisphere.

**Fig. 7:**
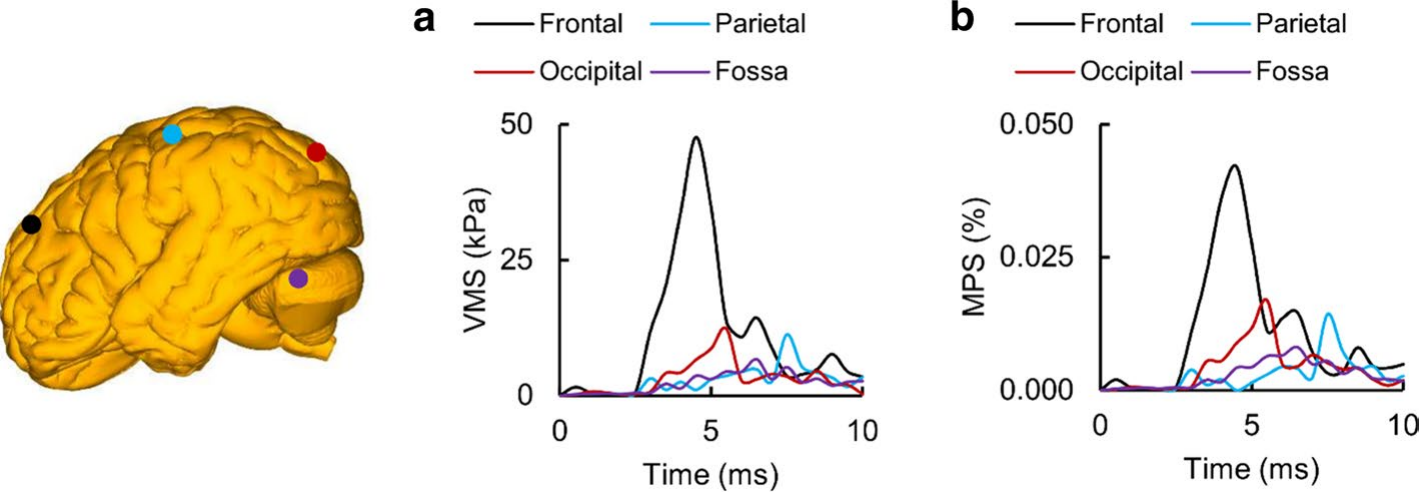
Simulations showing the variation of the von Mises stress (VMS) and the maximum principal strain (MPS) as a function of time and brain location in Nahum’s experiment [48]. Temporal profile of **a** the VMS and **b** the MPS at the frontal lobe, parietal lobe, occipital lobe, and fossa based on simulations using the detailed-vasculature model (Locations selected for comparison are indicated by colored circles on the brain geometry)

For Nahum’s experiment, we evaluated the temporal variation of the VMS and the MPS at four locations: (1) the frontal lobe sulcus, (2) the parietal lobe sulcus, (3) the occipital lobe, and (4) the fossa. Initially, we observed that the VMS and the MPS increased with time, with varying rates at each of these locations, and then decreased with time (Fig. 7a, b). In particular, the brain-tissue stresses and strains increased more rapidly in the anterior and posterior brain (i.e., the frontal lobe sulcus, the occipital lobe, and the fossa) than in the mid-brain (i.e., the parietal lobe sulcus). The peak VMSs and MPSs in the frontal lobe sulcus, parietal lobe sulcus, occipital lobe, and fossa were observed at 4.5 ms, 7.5 ms, 5.5 ms, and 6.5 ms, respectively. While the peak values of the VMS and MPS in the frontal lobe sulcus were higher than the corresponding values in the fossa by 609% and 417%, respectively, the peak brain-tissue stresses and strains were comparable in the parietal lobe sulcus and the occipital lobe.

**Fig. 8:**
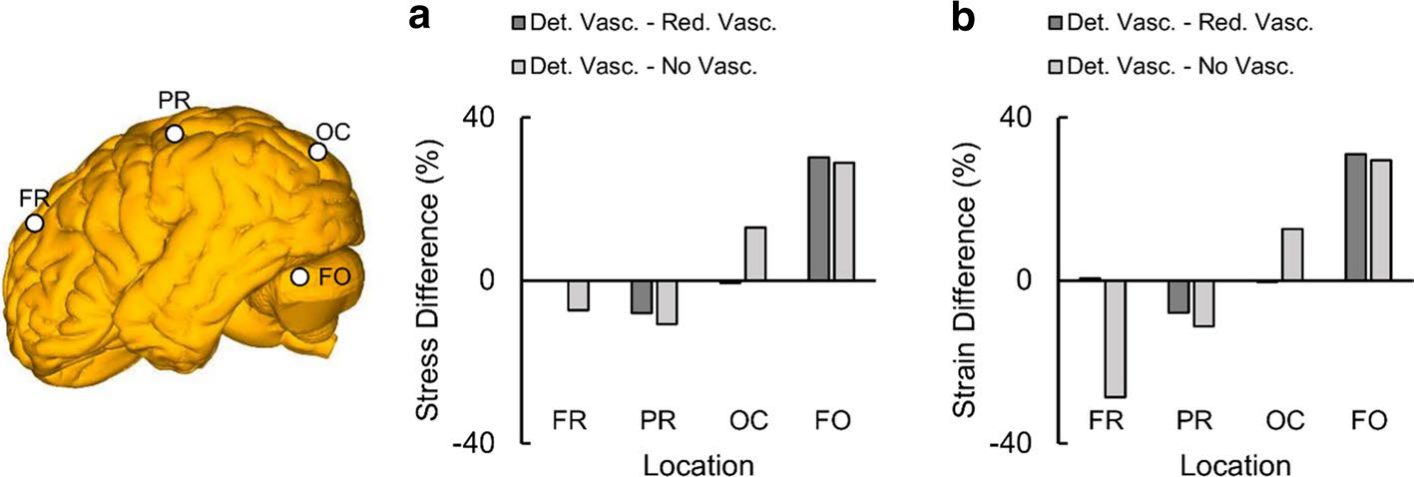
Differences in the peak von Mises stress (VMS) and the peak maximum principal strain (MPS) between models in Nahum’s experiment [48]. Bar graphs comparing **a** the peak VMS and **b** the peak MPS between the detailed- and reduced-vasculature models and between the detailed- and no-vasculature models. *Det. Vasc.* detailed-vasculature model, *Red. Vasc.* reduced-vasculature model, *No Vasc.* no-vasculature model, *FR* frontal lobe, *PR* parietal lobe, *OC* occipital lobe, *FO* fossa (Locations selected for comparison are indicated by white circles on the brain geometry)

We also compared the peak VMS and MPS at these four locations computed by the detailed-vasculature model with those obtained by the reduced-vasculature and no-vasculature models (Fig. 8). The peak VMS and MPS values in the frontal lobe were comparable for the detailed- and reduced-vasculature models but 7% and 28% higher, respectively, for the no-vasculature model. Compared to the detailed-vasculature model, the VMS and MPS values in the parietal lobe were 8% and 11% higher in the reduced- and no-vasculature models, respectively. The peak VMS and MPS values in the occipital lobe were comparable for the detailed- and reduced-vasculature models but 13% higher for the no-vasculature model. The peak VMS and MPS values in the fossa were comparable for the reduced- and no-vasculature models, being 31% and 29% lower, respectively, than the peak VMS and MPS of the detailed-vasculature model.

**Fig. 9:**
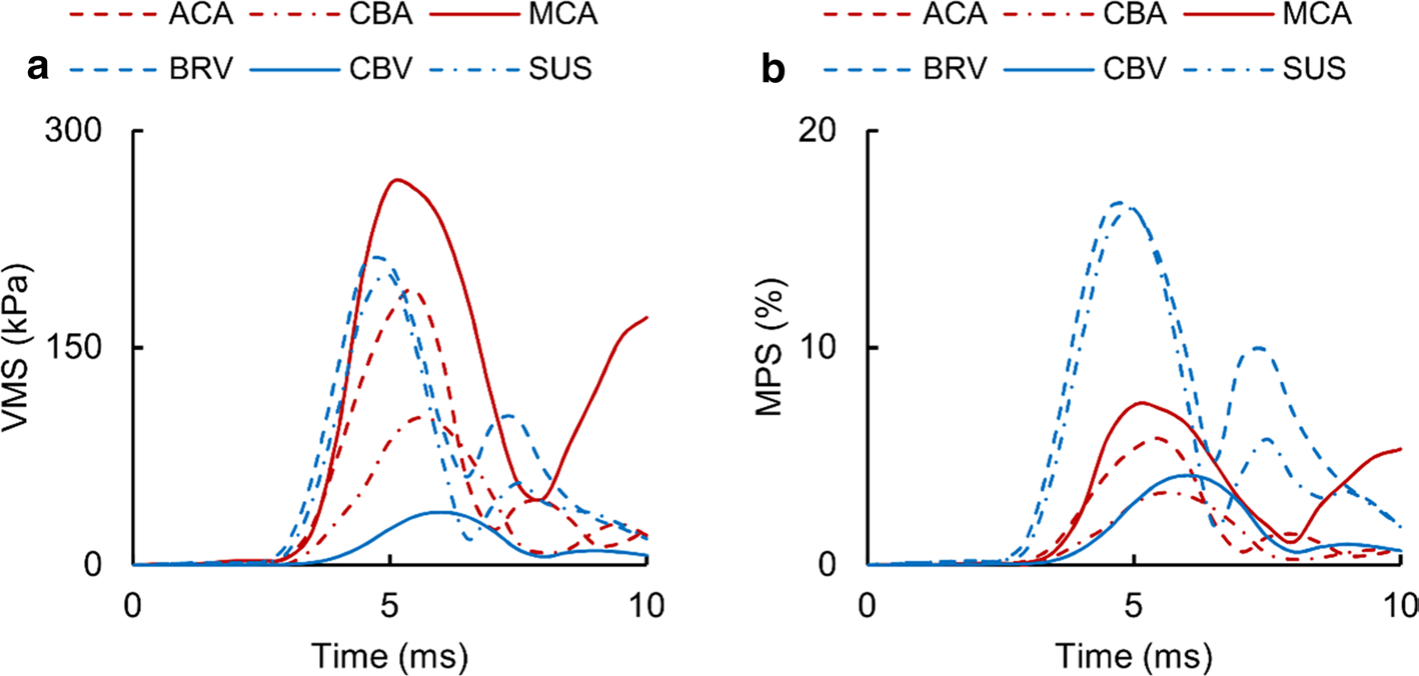
Temporal variation in **a** von Mises stress (VMS) and **b** maximum principal strain (MPS) for the cerebral vasculature in Nahum’s experiment [48]. *ACA* anterior communicating artery, *CBA* cerebellar artery, *MCA* middle cerebral artery, *BRV* bridging vein, *CBV* cerebellar vein, *SUS* superior sagittal sinus

For the detailed-vasculature model in Nahum’s experiment [48], we also evaluated the temporal variation in the VMS and MPS values for the cerebral vessels (Fig. 9). The stresses and strains increased more rapidly for vessels located proximal to the frontal lobe (i.e., the anterior cerebral artery, the middle cerebral artery, the sagittal sinus, and the bridging vein) than for vessels located distal to the frontal lobe (i.e., the cerebellar artery and the cerebellar vein). Accordingly, we observed peak VMS and peak MPS for the proximal and distal vessels at 5 ms and 6 ms, respectively. The peak VMS for the middle cerebral artery was 159% and 618% higher than the corresponding value for the cerebellar artery and the cerebellar vein, respectively. The peak VMS was comparable for the anterior cerebral artery, the bridging vein, and the sagittal sinus. The peak MPSs were identical for the bridging vein and the sagittal sinus. The peak MPS was comparable for the middle cerebral artery and the anterior cerebral artery; it was also comparable for the cerebellar artery and the cerebellar vein.

## Discussion

In this study, we developed a high-fidelity 3-D FE model for simulating the biomechanical effects of blunt trauma on the human head. We simulated three blunt trauma experiments and validated our model predictions by comparing the simulated and the experimentally measured ICP and RD at different locations within the brain. By including the detailed network of cerebral vessels, we can more precisely account for brain-tissue stiffness and the resulting redistribution of blunt-induced stresses and strains, thereby enhancing the accuracy of the model predictions. To assess this enhancement for blunt loading, we compared and contrasted the model-predicted ICP, VMS, and MPS values with those obtained from a reduced-vasculature model and a model with no vasculature for the same loading conditions.

### Model validation

For Nahum’s experiment, we observed good agreement between the model-predicted and the experimental ICP values, with peak-pressure discrepancies of less than 10% and temporal phase-shift differences of less than 0.5 ms (Fig. 4b). Previous model validations have reported similar comparisons between simulations and experiments. For example, Zhao et al. [21] observed peak-pressure discrepancies of less than 15% and Kleiven and von Holst [23] reported temporal phase-shift differences of less than 0.5 ms. In contrast, Levadnyi et al. [59] reported a phase shift of 1.0 ms and Yang et al. [60] observed peak-pressure discrepancies of 18%. The larger phase-shifts and peak-pressure discrepancies in these studies could be due to differences between the simulated and experimentally measured force. In addition, the ICP values depend on the neck boundary condition and the constraint between the brain and the subarachnoid space. We used a free neck boundary condition (Fig. 3a), which resulted in translation of the head. Chen and Ostoja-Starzewski [7] compared the free and fixed boundary conditions and showed that, while the free boundary condition does not result in a lower ICP, the fixed boundary condition causes both head rotation and a reduction in the predicted ICP by as much as 56%. We constrained the relative motion between the brain and the subarachnoid space, which resulted in a good match between the simulated and experimental ICP values. This is consistent with the study by Kleiven and Hardy [13] who compared the tied constraint with a sliding condition and showed that the tied constraint yields more accurate results. We did not observe any differences in the ICP values for the detailed-, reduced-, and no-vasculature models, consistent with previous studies [28, 42]. This is because while the inclusion of vasculature increases the brain stiffness (i.e., the shear modulus), it does not change its compressibility (i.e., the bulk modulus) [42].

For Trosseille’s experiment, we observed good agreement between the simulated and experimental ICP values in the frontal lobe and lateral ventricle, with peak-pressure discrepancies of less than 23% and temporal phase-shift differences of less than 1.4 ms (Fig. 5b). Previous model validations have reported similar comparisons between the simulated and experimental ICP values. For instance, Willinger et al. [45], Zhao et al. [21], and Mao et al. [15] observed peak-pressure discrepancies of less than 19% and temporal phase-shift differences of less than 1.3 ms. The peak-pressure discrepancy in the third ventricle (33.7%) was also comparable to the values reported by Zhao et al. and Khanuja and Unni [12], who observed a peak-pressure discrepancy equivalent to 32%. We applied the force derived from the measured acceleration on the model forehead, similar to the study by Khanuja and Unni, which resulted in a difference of 41% between the simulated and experimental ICP values in the occipital lobe. In contrast, Mao et al., Willinger et al., and Zhao et al. reported peak-pressure discrepancies equivalent to 361%, 362%, and 260%, respectively. The higher peak-pressure discrepancies reported in these studies could be possibly attributed to the subtractive effect arising from the application of the measured acceleration to the center of gravity of the model [61].

For Hardy’s experiment, we observed good agreement between the model-predicted and experimental RD values in the Y direction, with peak-displacement discrepancies of 27% in the right cerebral hemisphere (Fig. 6b) and 24% in the left cerebral hemisphere (Fig. 6d). Our results were consistent with other models in terms of the temporal profiles of the RD but differed in terms of the peak RD values. For example, while the peak-displacement discrepancy corresponding to NDT-4 was higher than those observed by Mao et al. [15] and McAllister et al. [62], the discrepancy corresponding to NDT-11 was lower than those observed in their models. This discrepancy could be possibly attributed to the selection of the brain shear modulus and the brain–skull constraint. The brain shear modulus in our model and the model developed by McAllister et al. was 75% and 140% higher, respectively, than the value reported by Mao et al. Besides, Mao et al. constrained the relative motion between the brain and the skull, consistent with our study, whereas McAllister et al. assumed a sliding constraint between the skull and the brain. However, Kleiven and Hardy [13], who compared the RD values for different brain-tissue properties and constraints between the brain and the skull, reported that while the RD values predicted by the tied and sliding constraints differed by less than 15%, compliant brain-tissue properties increased the RD values by as much as 720%. These observations indicate that the differences in the predicted RD values between our model and the models developed by Mao et al. and McAllister et al. could have possibly resulted from the selection of the brain-tissue properties.

### Brain and vasculature stresses and strains

Overall, our blunt loading simulations for Nahum’s experiment showed that the VMS and the MPS initially developed in the frontal and occipital lobes and moved into the parietal lobe as time progressed (Fig. 7). They also showed that the inclusion of additional vasculature redistributes the brain-tissue stresses and strains by as much as 30% (Fig. 8). For the detailed-vasculature model, we observed higher VMS in the frontal lobe as compared to the occipital lobe (Fig. 7a), consistent with the work of Claessens et al. [63]. We observed peak VMS values equivalent to 84.0 kPa and 67.0 kPa in the corpus callosum and brainstem, respectively, which were within the range of values reported previously [11, 12]. For example, Khanuja and Unni [12] observed a peak VMS equivalent to 66.0 kPa in the brainstem, whereas Horgan and Gilchrist [11] reported a peak VMS value in the corpus callosum that ranged from 2.4 to 250.0 kPa.

For the detailed-vasculature model in Nahum’s experiment, we observed higher MPS in the frontal lobe as compared to the occipital lobe (Fig. 7b). This is consistent with the work of Zhang et al. [28], who reported higher MPS values in the frontal cortex as compared to the occipital cortex for blunt loads that generate translation of the head. The localized reduction in the MPS depends on the amount of vasculature. For example, inclusion of the bridging veins reduced the peak MPS in the reduced-vasculature model by 8% as compared to the no-vasculature model, and inclusion of the middle cerebral arteries in the detailed-vasculature model reduced the MPS in the parietal lobe by an additional 3%. The reduction in the MPS resulting from the inclusion of vasculature was also consistent with other human head FE models [28–30]. For instance, Ho and Kleiven [30], who modeled only the major cerebral veins and arteries, reported a strain reduction equivalent to 8%. In contrast, our detailed-vasculature model, which considers a more comprehensive network of cerebral vessels, predicted strain reductions by as much as 28%.

Interestingly, we observed larger differences in the stresses and strains in the fossa when we compared the detailed-vasculature model with the reduced-vasculature model (Fig. 8). We attributed these large differences to the amount of additional cerebellar vasculature represented in the detailed-vasculature model, including the cerebellar veins and the cerebellar arteries. In contrast, as the amount of vasculature in the frontal, parietal, and occipital lobes was comparable in both models, we observed smaller differences in the stresses and strains at the locations selected for comparison.

Overall, our blunt trauma simulations for Nahum’s experiment showed that the vasculature VMS and MPS developed initially in the vessels located proximal to the frontal lobe and then moved into the distal vessels as time progressed (Fig. 9). They also showed that the peak VMS for the arteries was as much as 618% higher as compared to the veins. Our results differed from other models in terms of the temporal profiles and peak values of the MPS. For example, while we observed the MPS in the anterior cerebral artery to initially increase and then decrease, Zoghi-Moghadam and Sadegh [64] observed the strain to increase linearly. Furthermore, unlike this model, the vessel strain in our model did not exceed the failure limit. This discrepancy could be due to their assumption of linear elasticity in the vasculature, the limited amount of vasculature represented in their model, methodological differences in the simulations of the blunt load between the two models, or all of the above.

### Study limitations

Our study has limitations. First, we evaluated the influence of vasculature only for head translation. However, as the redistribution of MPS values resulting from the inclusion of vasculature is consistent for both translation and rotation [28, 30], we expect our general findings to hold for scenarios involving rotation of the head. Second, we did not model cerebral veins and arteries smaller than diameters of 0.52 mm and 0.24 mm, respectively, and assigned a uniform thickness for the vessel wall. We believe that the inclusion of vessels with a diameter smaller than 0.52 mm for veins and 0.24 mm for arteries would change the brain-tissue stiffness, and subsequently modify the values of the RD, VMS, and MPS. However, we do not expect to observe changes in the model-predicted ICP values, because the inclusion of vasculature does not change the brain-tissue compressibility. In addition, while we believe that the assumption of uniform wall thickness could possibly influence the magnitude of the vascular-tissue stresses and strains induced by the blunt impact, we expect our overall findings to remain valid.

Third, as our study primarily focused on the brain-tissue stiffening response arising from the inclusion of the cerebral vasculature, we used the embedded element method to represent the effect of the cerebral vessels, excluded the blood, and did not specify the blood pressure in our FE model, consistent with previous studies [29, 42]. Moreover, as the peak coup pressures for the inclined frontal impact (147.9 kPa) and the normal frontal impact (87.5 kPa) were higher than the systolic arterial blood pressure (12.0 kPa) [53] by 1133% and 629%, respectively, we do not believe that the inclusion of blood pressure would significantly change the model-predicted ICP values. Fourth, the embedded element method could potentially add mass to the model [65]. However, based on our calculations, the additional mass resulting from the vasculature was only 0.06% of the total mass of the human head, implying that the potential effect of the added mass was insignificant. Fifth, we approximated the meninges as a hyperelastic solid that could be alternatively modeled as a fluid [66]. Nonetheless, our assumption resulted in good correspondence between the model-predicted and the experimentally measured ICP. Finally, we assumed homogeneous properties for the skin and necessarily excluded mechanical properties specific to the face and scalp tissues [67], which can possibly influence the biomechanical responses for offset impacts [68]. Nonetheless, our assumption resulted in good correspondence between the model-predicted and the experimentally measured RD.

These limitations notwithstanding, we believe that our 3-D high-fidelity human head FE model advances TBI research related to dynamic impacts and impulses to the head. By increasing model fidelity, we expect to enhance our ability to identify correlates between predicted biomechanical responses and observed injuries resulting from blunt trauma to the head.

## Conclusions

To conclude, we developed a high-fidelity 3-D FE model of the human head and characterized the biomechanical responses of the brain to blunt loading. In the FE model, we explicitly represented the detailed network of superficial and embedded cerebral veins and arteries. Our study showed that such a detailed representation of the cerebral vasculature was the key attribute of our model, influencing the shear response and resulting in the redistribution of stresses and strains in the brain tissues by as much as 30%. The high-fidelity model developed in this study could be helpful in correlating predicted biomechanical responses with observed brain injuries and in predicting injury thresholds for blunt impact.

## Data Availability

All data generated or analyzed during this study are included in the article.

## Abbreviations

3-D: Three-dimensional
CDC: Centers for Disease Control and Prevention
FE: Finite element
ICP: Intracranial pressure
MPS: Maximum principal strain
RD: Relative displacement
TBI: Traumatic brain injury
VMS: Von Mises stress
